# Massive air embolism as a complication of upper gastrointestinal endoscopy: A case report illustrating a stroke mimic, literature review, and suggested management

**DOI:** 10.1002/ccr3.1725

**Published:** 2018-08-08

**Authors:** Anders Kjellberg, Harriet Nyström, Martin Söderberg, Aldona Dlugosz, Henrik Jörnvall, Anna Steinberg

**Affiliations:** ^1^ Perioperative Medicine and Intensive Care Karolinska University Hospital Stockholm Sweden; ^2^ Department of Neuroradiology Karolinska University Hospital Stockholm Sweden; ^3^ Department of Medicine Huddinge Center for Digestive Diseases Karolinska Institutet Karolinska University Hospital Stockholm Sweden; ^4^ Department of Clinical Neuroscience Karolinska Institutet Stockholm Sweden; ^5^ Department of Neurology Karolinska University Hospital Stockholm Sweden

**Keywords:** air embolism, arterial air embolism, brain air embolism, cerebral air embolism, cerebral arterial gas embolism, gas embolism, hyperbaric oxygen treatment, hyperbaric oxygen, upper gastrointestinal endoscopy

## Abstract

Cerebral air embolism should be considered in case of stroke symptoms during any invasive procedure. Transport to a hospital with neurosurgical/hyperbaric oxygen treatment (HBOT) facility could improve the outcome for the patient. Absence of air on computed tomography (CT) scan should not disqualify a patient from HBOT if air embolism is suspected.

## 1 INTRODUCTION

Cerebral arterial gas embolism (CAGE) is a rare complication but can occur after many common invasive medical and surgical procedures including upper endoscopy.^1^, ^2^, ^3^, ^4^, ^5^, ^6^, ^7^, ^8^ Cerebral air embolism due to upper endoscopy without major vessel injury is extremely rare.^7^ Hyperbaric oxygen treatment (HBOT) is the only definite treatment for CAGE.

The purpose of this article was to highlight this unusual, but potentially lethal, complication of upper gastrointestinal endoscopy. We present a case of severe cerebral air embolism with initial unconsciousness and surprisingly good outcome correlated to repeated HBOT and 2 weeks of diligent neurointensive care.

## 2 CASE REPORT

A 42‐year‐old healthy man, with a previous episode of food impaction and increased number of eosinophils in esophageal biopsies taken during an index upper endoscopy 6 weeks earlier, underwent outpatient control gastroscopy after receiving proton pump inhibitor treatment. The upper endoscopy was performed under conscious sedation with midazolam, with a standard video endoscope and the air pressure setting on the video processor at “medium.” Biopsies from distal, middle, and proximal esophagus were taken according to standard protocol. At the end of the 7‐minute‐long procedure, just after biopsies from proximal esophagus was taken, the patient's heart rate decreased to 46, the oxygen saturation decreased to 90%, and he developed generalized tonic‐clonic seizures. The endoscope was rapidly withdrawn, and the oxygen saturation and heart rate normalized spontaneously. However, the patient was unresponsive despite reversal with flumazenil. While hemodynamically and respiratory stable, he remained unresponsive, Glasgow Coma Scale (GCS 3), with pupils midsize, equal, and reactive to light, and a negative Babinski sign.

**Figure 1:**
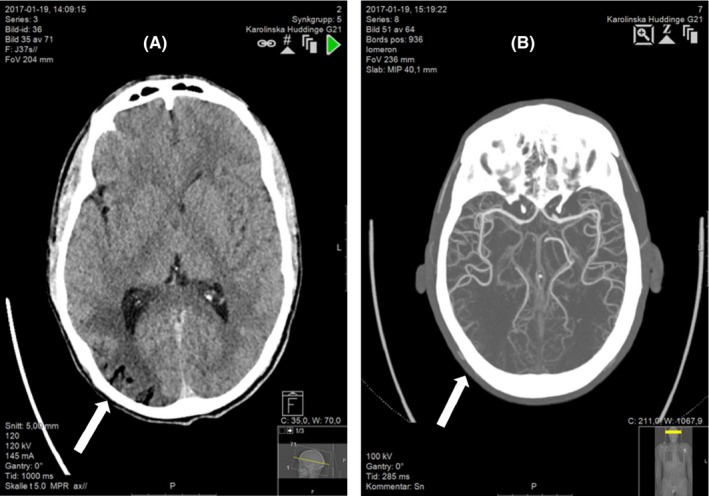
A, The scan showed gyriform air bubbles within the cortical arteries most pronounced in the fronto‐parieto‐occipital region in the right hemisphere, but also in the frontoparietal areas in the left hemisphere. The pattern was almost that of cortical watershed areas bilaterally, but most dominant on the right posterior side. B, The cervical and brain computed tomography angiography (CT‐AI) 70 min after the insult showed disappearance of air bubbles and no vessel occlusion. Noteworthy, but not stated in the initial report, there was a general thinning of arteries in the distal parietal areas, probably caused by an early reaction of the endothelial cells with swelling of the intima and narrowing of the arteries most severely affected by large bubbles of trapped air, as previously seen on the CT scan one hour earlier

An immediate computed tomography (CT) scan 30 minutes postinitial symptoms revealed massive air embolism in the right hemisphere. (Figure 1A). The radiologists first suspected infarctions, which was later modified. A complementing CT angiography (CT‐AI) was performed 70 minutes after the initial CT scan to rule out differential diagnoses, and the delay was due to recurrent seizure and intubation. The most striking finding was complete resolution of air in the vessels. (Figure 1B).

**Figure 2:**
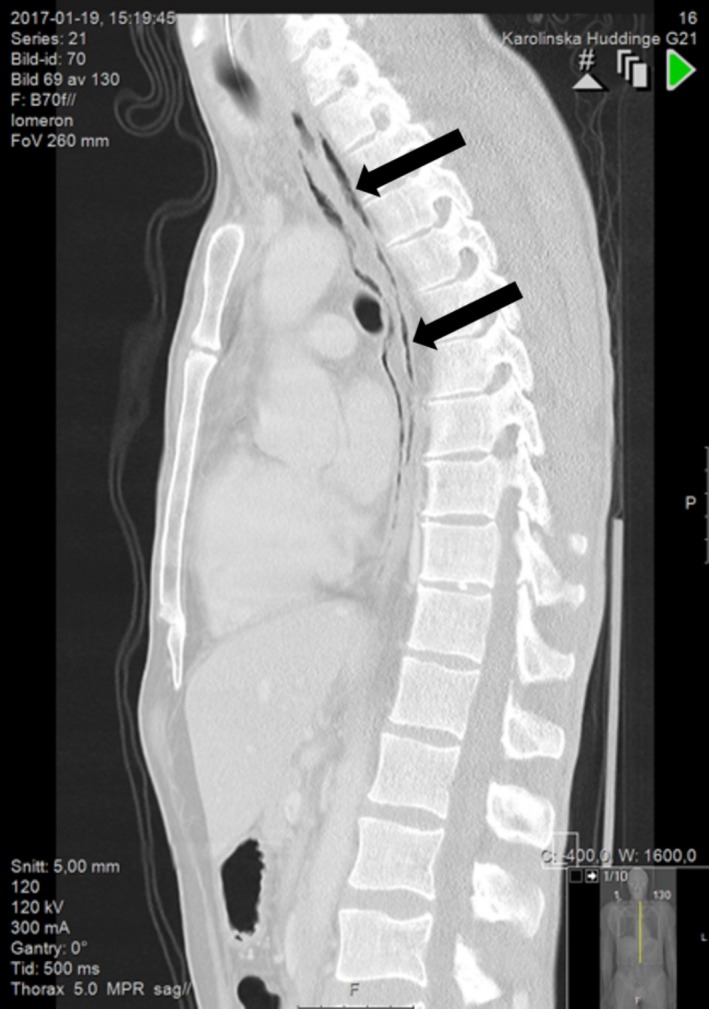
Free air in the mediastinum around the esophagus

Simultaneously with the CT‐AI, a CT of the thorax was performed showing air around the whole length of the esophagus and small amounts of free air in the mediastinum behind the left atrium. (Figure 2).

**Figure 3:**
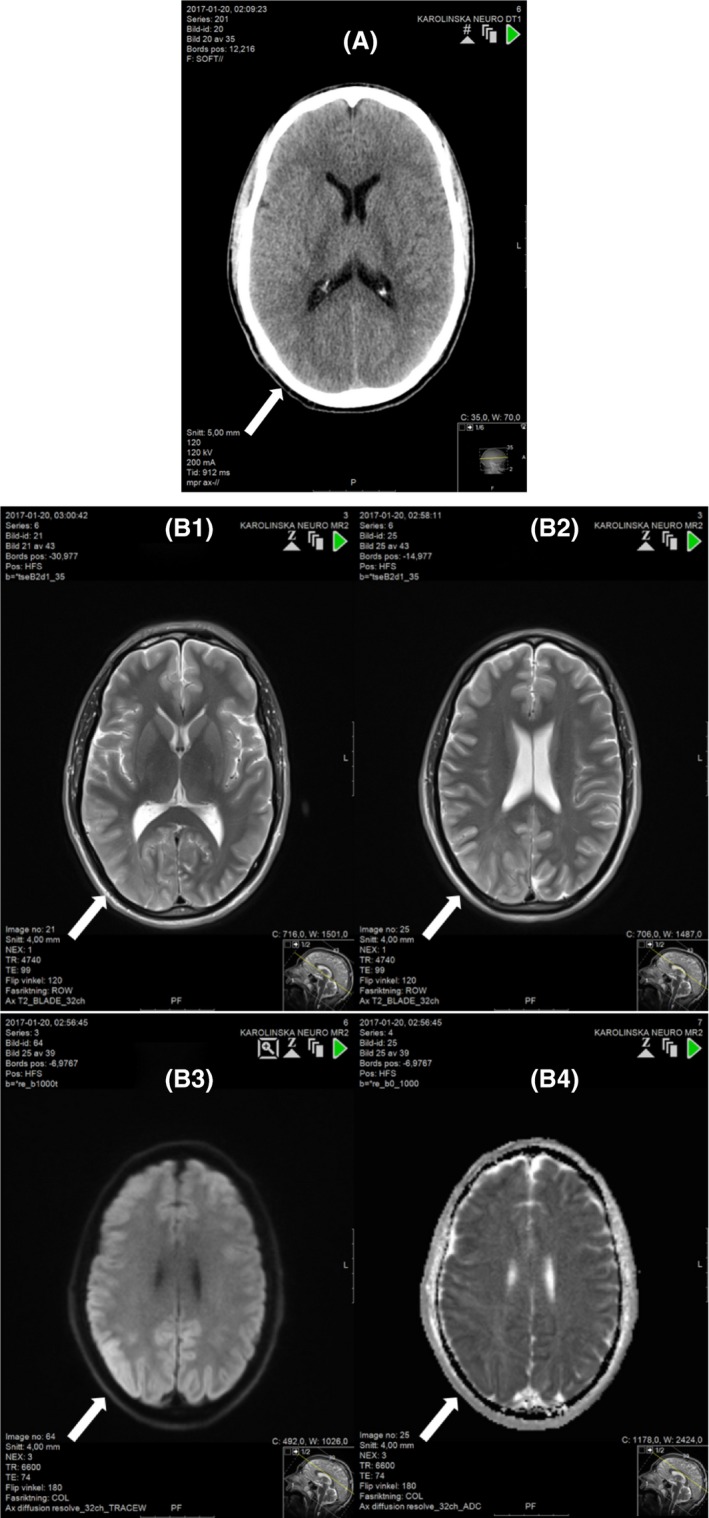
A computed tomography CT scan 12 hours after initial CT showing normalisation of attenuation and only slight swelling in the right parietal area. (A). T2 weighted images in MRI is almost normal 14 hours after initial CT, only diffuse high signal in cortical areas and white matter in the posterior right hemisphere (1‐2). Diffusion weighted images showed restricted diffusion in large cortical areas, on the right side. The restricted diffusion was interpreted as ischemic areas, but with the important notion that the mechanism of air embolism is not the same as vessel occlusion (3‐4) (B).

The neurologist on call contacted the hyperbaric unit, and the patient was transported to the hyperbaric unit, arriving three hours after initial symptoms. HBOT with United States Navy (USN) (Table 6 (Figure 6A)) was commenced immediately. The patient remained completely unresponsive during the initial 20 minutes of compression despite a confirmed gas embolism, and to maximize the therapeutic effect, the treatment table was extended to 8 hours and 5 minutes. (Figure 6B). After initial HBOT, the patient underwent a second CT scan, 12 hours after the initial CT, which to our surprise showed normalization, with only slight reduced attenuation in the right parietal area (Figure 3A). As the patient remained unconscious and there was a concern of much more damage to the brain than evident on the CT, a magnetic resonance imaging (MRI) was performed. The indication for early MRI performed 14 hours after air embolism was to make an estimation of putative ischemic lesions with diffusion weighted imaging.

Diffusion weighted MRI indicated cytotoxic edema, predominantly in the areas previously showing the greatest amount of air embolism on the initial CT scan (Figure 3B). The findings should be interpreted with caution and could be partly reversible.

A wake‐up examination was performed, but since the patient remained GCS 3, neurosurgical monitoring was commenced with intracranial pressure (ICP) monitoring, microdialysis, and continuous electroencephalography (EEG). The patient was continuously monitored and received additional daily HBOT 2.8 ATA, 113 minutes for 4 days. ICP was stable until day 5, but then, ICP instability and suspicion of status epilepticus required treatment with pentothal at burst suppression until day 10. As the patient presented with seizures, levetiracetam was prescribed with an initial dose of 1000 mg 12 hourly. Levetiracetam concentration was checked and dose increased to 1250 mg 12 hourly due to low serum concentrations and suspected epileptic activity on EEG. After 2 weeks, the patient had no seizures, and during the rehabilitation phase, levetiracetam was discontinued.

**Figure 4:**
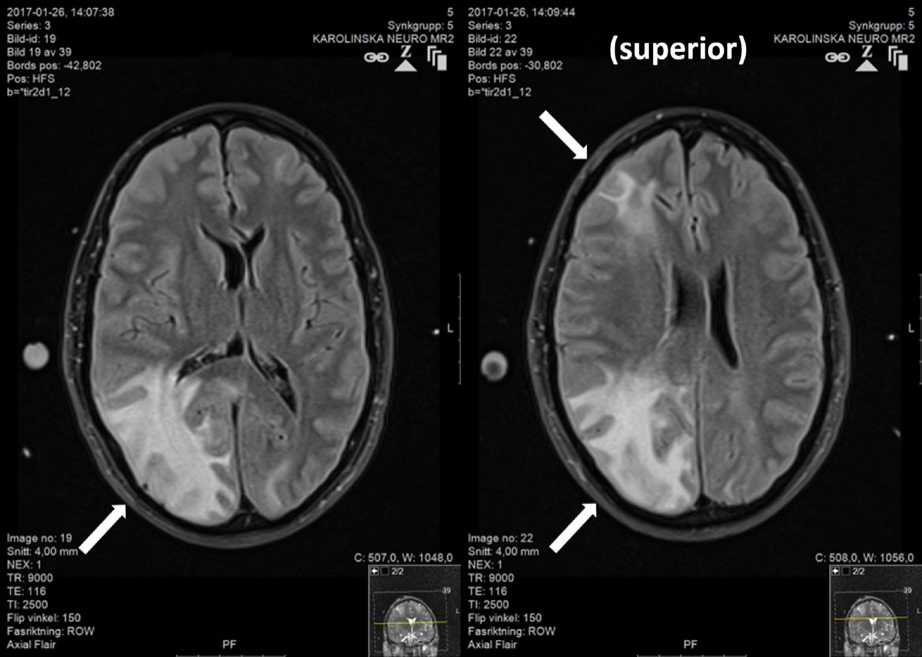
Second magnetic resonance imaging (MRI) after 1 wk (FLAIR images). There were large areas of vasogenic edema in mostly the white matter with expansive effect with compression of sulci over the hemispheres and a left midline shift of 4 mm to the left. There was also vasogenic edema in the superior areas of the brain

A second MRI was performed after 1 week showing progression with severe swelling bilaterally, but most pronounced in the white matter of the right hemisphere (Figure 4). During the hospital stay of 3 weeks, the patient was examined with extensive radiology. A series of four brain CT scans, one CT angiography of the brain and neck arteries, five CT of the thorax/esophagus/pulmonary arteries, and three MRI scans of the brain was performed together with a few chest X‐rays and ultrasounds. The initial CT finding with small artery and capillary air mimicking infarcts developed into large areas of reversible edema with swelling, and finally limited cortical infarcts with laminar necrosis and gliosis in the white matter.

### 2.1 Outcome

**Figure 5:**
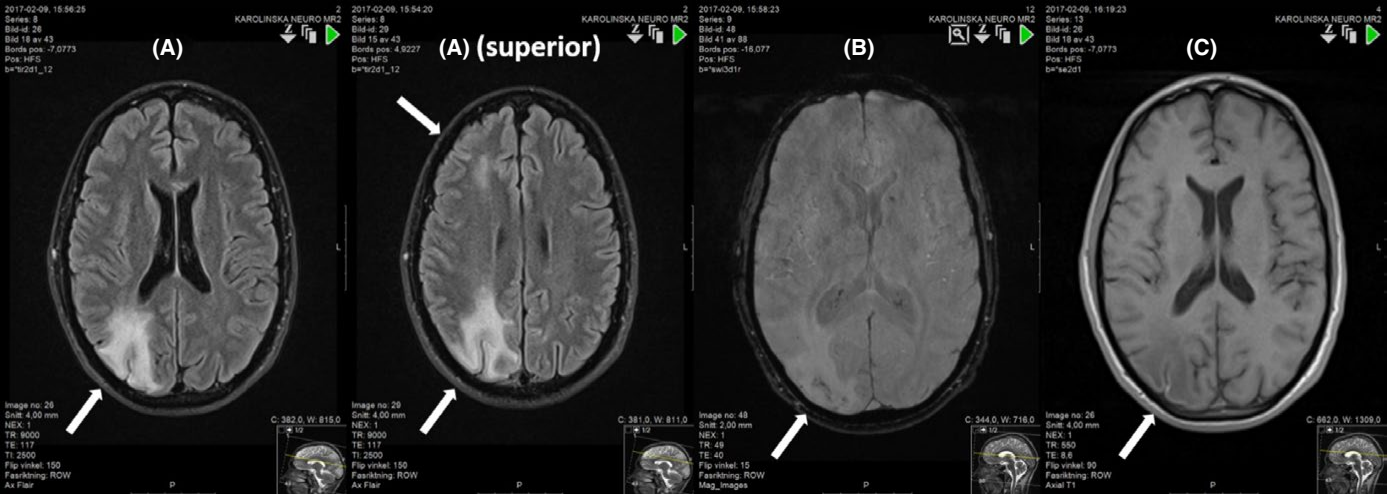
Third magnetic resonance imaging (MRI) after 3 wk (FLAIR images) when the patient was Glasgow Coma Scale (GCS 14) in the step down neurological ward. There was resolution of swelling, but widespread signal changes in the white matter in the right hemisphere interpreted as gliosis (A). The worst area of previous gyriform air also had small cortical microbleedings, (B). There were areas of cortical infarcts with laminar necrosis only affecting a thin cortical layer, and (C). The most affected areas were prefrontal, precentral gyrus, medial and posterior parietal lobe, and posterior lateral temporal and occipital lobe on the right side

Despite initial poor neurological presentation, the patient underwent a remarkably good recovery with a favorable outcome. ICP improved after 9 days; and pentothal infusion was stopped on day 11; neurology improved slowly; and on day 13, he was responsive and obeyed commands but with left hemiplegia. He was extubated on day 15 and then continuously improved until discharge from neurorehabilitation 3 months after the insult. The third and final MRI was performed after 3 weeks, when the patient was fully awake with a GCS score of 14. The finding was resolution of swelling and regression of edema (Figure 5). At discharge, he displayed only mild disability, slight neglect, and slight fine motor disability in the left hand. Eight months after the incident, he was back to work with minimal left spatial disability.

## 3 DISCUSSION

Iatrogenic gas embolism occurs in 2.65 per 100 000 hospitalizations and is associated with high long‐term mortality and morbidity and time to hyperbaric oxygen therapy of 7 hours or less reduce the risk of mortality and neurological sequelae. The presence of Babinski signs at the time of ICU admission is associated with poor long‐term outcome.^9^ Other studies have confirmed the association between short time (<6 hours) to HBOT and improved neurological outcome.^10^ Furthermore, gyriform air on initial CT scan, old age, initial conscious disturbance, and hemiparesis are correlated to unfavorable outcome.^11^

The most common reason for a massive air embolism is a persistent foramen ovale (PFO).^7^ In our patient, PFO could not be diagnosed with transthoracic echocardiography, and a transesophageal echocardiography was not conducted due to the risk of possible complications from an already injured esophagus. Once discharged, the patient was understandably reluctant to undergo further invasive examinations. As an alternative mechanism for air embolism, intrapulmonary shunts as well as transcapillary transfer of large air emboli have been proposed.^12^, ^13^ The mechanism of air embolization in our patient remains unclear.

The neurological deficit is initially caused by occlusion of arterioles leading to ischemia, but as shown in the radiology of our patient, the air is not present on the second CT scan. Secondary injury occurs due to ischemia/reperfusion (I/R), leading to a thromboinflammatory response in the affected area. HBOT has been shown to attenuate I/R injury in many different settings.^14^, ^15^

The indication for HBOT in air embolism has been attributed to Boyle's law, to reduce the size of the air bubbles. Emerging evidence of the anti‐inflammatory effects of HBOT was the reason for our decision to give a long initial treatment and to continue with additional treatments. Irrespectively of the mechanism of action of HBOT, we presume that it contributed to the remarkably good outcome of our patient despite very poor neurology and severe radiology findings.

## 4 CONCLUSION

The massive air embolism, prolonged unconscious state, and cytotoxic edema did not correlate with the outcome of our patient. It is thus important not to disqualify a patient from neurointensive care and HBOT despite discouraging initial neurology and radiological results otherwise suggesting a poor prognosis. However, current evidence suggests HBOT to optimally be commenced within 7 hours of the insult. Based on experience from our case, repeated HBOT and diligent neurointensive care might be beneficial, despite severe neurological symptoms persistent 2 weeks after the incident.

From previous experience and our current case, we would suggest the following recommendations: Immediately following any clinical suspicion of CAGE, 100% oxygen should be administered with simultaneous ventilatory and circulatory support, and neuroprotective treatment. A HBOT facility with intensive care capacity shall be contacted and transport logistics planned. If other differential diagnoses are suspected, a CT scan of the head can be conducted, but further diagnostic procedures should not delay transport to a HBOT facility. The absence of air on a CT scan should not disqualify a patient from HBOT, if air embolism is suspected.

## CONFLICT OF INTEREST

None declared.

## AUTHORSHIP

**Figure 6:**
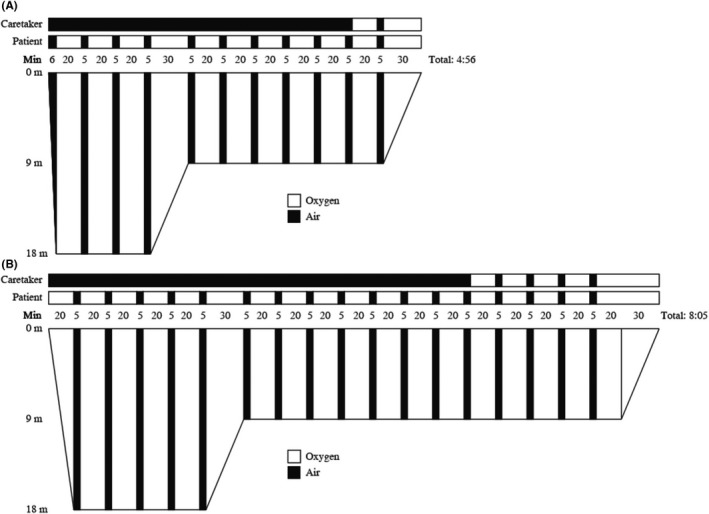
Standard treatment table 6 (A) and extended use during initial hyperbaric oxygen treatment (HBOT) of our patient (B)

AK and AS: wrote main part of the manuscript and submitted case report. AD: was the treating physician during the upper gastrointestinal endoscopy and provided information regarding the procedure. MS and AS: managed the patient in neurointensive care. MS: performed a literature search and provided information regarding the ICU stay. HN: performed initial radiology and provided images and statements from radiology. HJ: managed the patient during initial HBOT and provided Figure 6. All authors have reviewed and approved the final manuscript.
